# Effect of home-based lifestyle interventions on cognition in older adults with mild cognitive impairment: a systematic review

**DOI:** 10.1186/s12877-024-04798-5

**Published:** 2024-02-27

**Authors:** Cynthia Zou, Divinity Amos-Richards, Ram Jagannathan, Ambar Kulshreshtha

**Affiliations:** 1 Department of Family and Preventative Medicine, Emory University, Atlanta, USA; 2 Department of Neurology, Goizueta Institute, Emory University, Atlanta, USA; 3 Hubert Department of Global Health, Rollins School of Public Health, Emory University, Atlanta, USA; 4 Department of Epidemiology, Rollins School of Public Health, Emory University, Atlanta, USA

**Keywords:** Mild cognitive impairment, Lifestyle, Home-based, Web-based, Multi-component, Single-component

## Abstract

**Background:**

Mild Cognitive Impairment (MCI) is frequently a precursor to dementia, affecting aspects of cognition such as language, thinking, or memory. Lifestyle interventions are increasingly studied as potential means to slow the progression from MCI to dementia.

**Objective:**

A systematic review was conducted to investigate the effectiveness of home-based lifestyle interventions in reducing cognitive decline in older adults with MCI.

**Methods:**

A systematic review of randomized controlled trials (RCTs) was conducted to identify home-based lifestyle interventions for individuals with MCI from 1980 to 2023. These interventions were either single-component or multi-component and included diet, physical activity, stress-reduction, or cognitive stimulation treatments to assess their impact on cognition. We performed a comprehensive search in the PubMed, Web of Science, Google Scholar, Embase, and MEDLINE databases.

**Results:**

From 320 abstracts, 20 (6.25%) studies met the criteria for inclusion, with five multi-component and fifteen single-component studies. Eighteen home-based lifestyle interventions for MCI patients were focused on physical activity, diet, and/or cognitive training, while two studies were identified that incorporated stress reduction training as a method to improve cognitive function. Nineteen studies reported significant improvements in cognitive performance between the experimental and control groups post-intervention for at least one aspect of cognition. Four studies reported nonsignificant improvements in cognitive function between the two groups for at least one area of cognition.

**Conclusions:**

Home-based lifestyle interventions have the potential to improve cognition in elderly patients with MCI. However, future RCTs with larger sample sizes and longer intervention durations are needed to confirm these findings.

**Supplementary Information:**

The online version contains supplementary material available at 10.1186/s12877-024-04798-5.

## Introduction

Mild Cognitive Impairment (MCI) is an intermediate stage between normal aging and early dementia [1]. MCI is characterized by cognitive decline that can affect a person’s ability to perform everyday activities, such as the ability to remember important information or to attend appointments, complete complex tasks, understand written or verbal information, and problem-solving. MCI can be further categorized as amnestic MCI, which predominately affects memory, or as non-amnestic MCI, which affects areas of cognition other than memory. Globally, the prevalence of MCI among community-dwelling elderly aged 50 years and older is more than 15% [2]. Approximately 46% of people with MCI develop dementia within 3 years compared to only 3% of the similar age population [3]. Effective, non-pharmacological strategies to delay or prevent cognitive decline in older adults are an area of active research. Fortunately, evidence indicates that nonpharmacological interventions can improve cognition in older adults [4]. One meta-analysis of 28 studies and 2711 participants concluded that multidomain and nonpharmacological interventions were more strongly associated with improving memory, verbal fluency, executive function, and global cognition in older adults with MCI than single-domain interventions [4]. Traditionally, cognitive interventions have been in-person or group-based at a designated study location, such as a clinic. However, this usually requires a time commitment from participants to travel to the study site, and clinic logistics of space and personnel. 17% of elderly patients over the age of 65 are home-bound and unable to access services to support their health [5].

Home-based interventions have the potential to offer multiple advantages for participants compared to in-person approaches, such as reduced costs in terms of traveling, more accessibility, and potentially more anonymity [6]. Home-based interventions are feasible alternatives that would allow older adults the potential to improve their cognition from the convenience of their homes and have risen in prominence in recent years, especially during the COVID-19 pandemic [7]. However, there is sparse reviews or guidelines available that describes the effectiveness of home-based lifestyle interventions for patients with MCI. Therefore, the purpose of this systematic review is to identify the prevalence and effectiveness of home-based lifestyle interventions (i.e., diet, physical activity, stress reduction, cognitive training) on cognitive function in older adults with MCI.

## Methods

### Study selection criteria and search strategy

Randomized controlled trial (RCT) studies that included participants with MCI were included in this systematic review. The inclusion criteria also required that the interventions have lifestyle-based components, specifically diet, physical activity, stress reduction, or cognitive training. PubMed, Web of Science, Embase, Google Scholar, and MEDLINE were the databases used the complete this search. Studies from 1980 to 2023 were included. The year 1980 was set as a lower limit because the term “MCI” was introduced in the 1980s by Reisberg and colleagues to identify the transitional cognitive stage from normal aging to dementia [8]. We restricted our analyses to those published in English. The databases were searched using the following keywords: “mild cognitive impairment,” cognition, “cognitive function”, “physical activity,” “board games,” diet, stress, AND (RCT OR randomized OR controlled trial OR intervention) AND (home-based OR web-based). Interventions were classified as home-based if they partially or wholly occurred at the participant’s home. RCTs that evaluated consequences of lifestyle interventions on cognition were included. We also included studies that were pilot in nature.

Records were identified via database searching of the previously mentioned keywords and duplicates were removed. All titles and abstracts were analyzed for potential eligibility during the first round of screening. Papers were included if they (1) examined the effect of at least one type of lifestyle intervention (diet, physical activity, stress reduction, or cognitive training) on cognition, (2) included only individuals with MCI, (3) were RCTs, (4) were conducted from 1980 to 2023, and (5) had the intervention carried out at patients’ homes. Individuals with dementia or Alzheimer’s were excluded from this review, as well as study protocol articles. All potentially eligible articles were then retrieved in full text and further included or excluded based on the criteria mentioned above. Twenty studies met our eligibility criteria and were included in this review. In addition, data on the year of publication, setting and population, intervention and control groups, total sample size, outcome of intervention, and *p*-values were extracted to provide supporting information for this review. All data were independently extracted and assessed by two authors to minimize bias. Any discrepancies involved in selecting the studies to be included in this review were resolved by consensus or by an adjudicator (Dr. Kulshreshtha) if needed. Study information was extracted and stored in EndNote. Results of the literature search were tabulated and displayed using a PRISMA Flow Diagram. Details of individual studies included in the review were displayed via a table. Meta-analysis was not employed because studies were too dissimilar to each other in terms of sample size, study population, and intervention components. Sensitivity analyses were also not utilized in this review. The study adheres to PRISMA guidelines (http://www.prisma-statement.org/) for reporting systematic reviews.

## Results

**Fig. 1:**
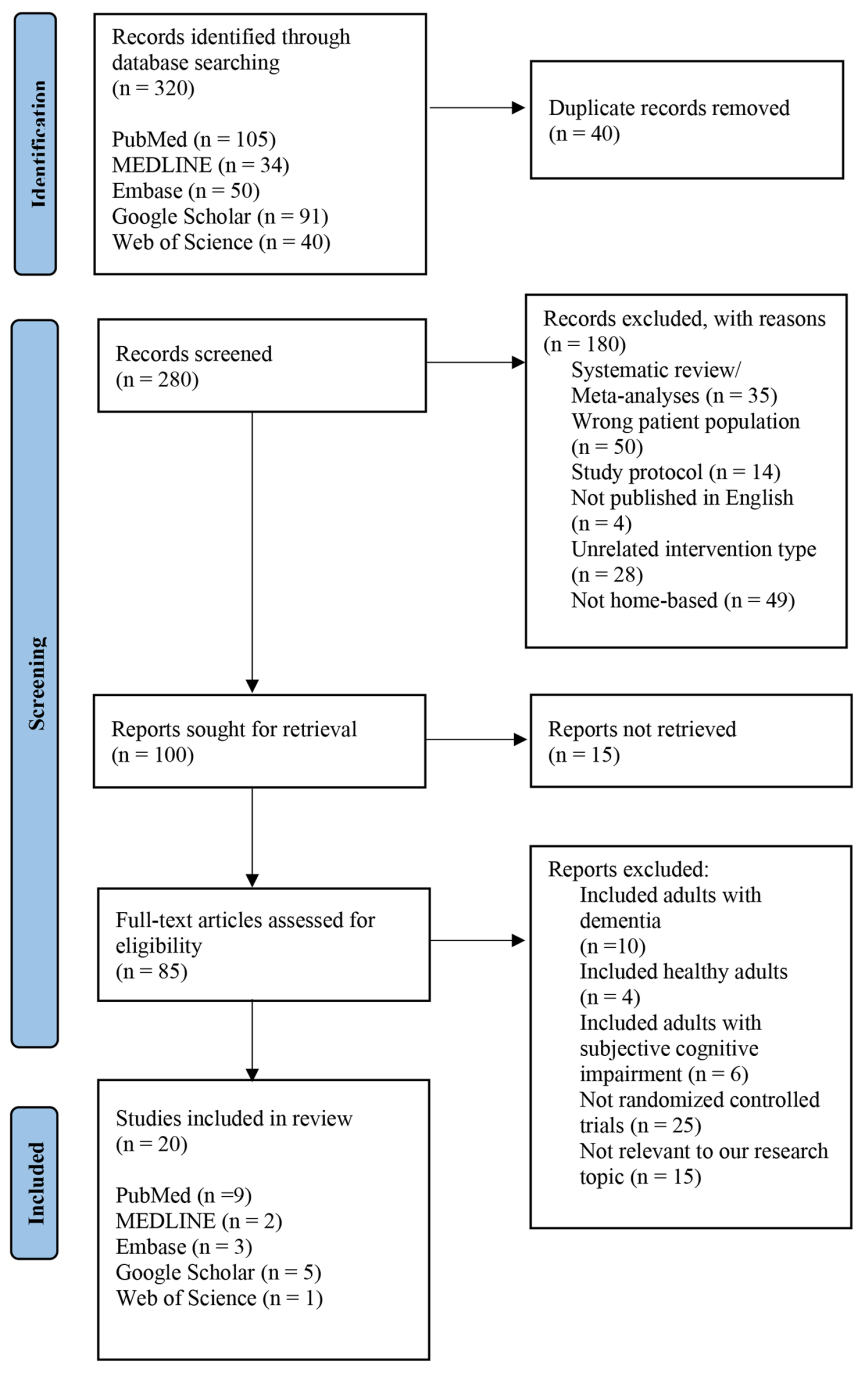
PRISMA Flow Diagram

Figure 1 presents the literature search and study selection process, resulting in twenty studies from different countries being included in the final analysis. Five of these were multi-component and fifteen were single-component studies. Table 1 shows specific details about each study included in this systematic review, including the population and setting, intervention duration and type, total sample size, control groups, outcomes, and *p*-values. Two studies focused on MCI individuals with specific clinical circumstances (i.e., HIV or obesity) [9, 10]. Furthermore, three studies focused exclusively on individuals with amnestic mild cognitive impairment (aMCI) [11–13]. Individual studies were evaluated for bias and study quality was graded by reviewers.

**Table 1:** Study Characteristics

Study	Setting and Population	Intervention Description	Control Group	Intervention Duration	Total Sample Size		Intervention Type(s)		Outcome and *P*-values		
Frain et al. (2018) [9]	Adults 50 or older with HIV and MCI in USA	Home-based computerized cognitive training program	Health-related newsletter *via* email and follow-up phone calls	8 weeks	24	Cognitive training: BrainHQ, a product that provides brain-training exercises to improve cognitive performance, was used		Cognitive improvement was seen immediately after the intervention Improvement in intervention group on total MoCA scores compared to control: *p* = 0.04			
Horie et al. (2016) [10]	Obese patients with MCI, aged 60 or older in Brazil	Conventional medical care and nutrition counseling	Conventional medical care	12 months	80	Physical activity: Encouraged to engage in at least 150 min of moderate-intensity aerobic physical activity weekly Diet: Nutrition counseling aimed to promote healthy eating habits and weight loss through caloric restriction		Intentional weight loss through diet is associated with cognitive improvement Global cognition: *p* < 0.0001			
Jeong et al. (2016) [11]	Patients aged 50–85 with aMCI from nationwide hospitals in South Korea	1)Group-based cognitive intervention (GCI) 2)Home-based cognitive intervention (HCI)	Did not receive either intervention	12 weeks	293	Cognitive training: GCI: comprehensive multimodal cognitive intervention HCI: Homework materials with tasks for memory and other cognitive domains		The GCI and HCI resulted in cognitive improvements in aMCI Compared to controls: Subjects in GCI: *p* = 0.01 Subjects in HCI: *p* = 0.02			
Sungkarat et al. (2017) [12]	Adults ages 60 and older with aMCI were recruited from a local community in Chiang Mai, Thailand	Center-based and home-based Tai Chi training	Received educational material about cognitive impairment and fall prevention	3 weeks center-based and 12 weeks home-based Tai Chi (15 weeks total)	66	Physical activity: Tai Chi exercises		Compared to control group: Significantly improved performance on Logical Memory, Block Design, and TMT (B–A) Logical memory: *p* = 0.006 Block design score: *p* = 0.01 Trail-making Test Part B-A score: *p* = 0.005			
Suzuki et al. (2012) [13]	Older adults with aMCI ages 65 to 93 years at a community center in Japan	Multicomponent exercise group	Education about health control group	12 months	50	Physical activity: Daily home-based exercise, structured exercise program, outdoor walking		Improvements in cognitive function for treatment group (group × time interactions for the mini-mental state examination (*p* = 0.04), logical memory of immediate recall (*p* = 0.03), and letter verbal fluency test (*p* = 0.02))			
Manenti et al. (2020) [14]	Adults over 65 years old with MCI recruited in Italy	1)Face-to-face cognitive virtual reality rehabilitation system (VRRS) followed by home-based cognitive VRRS training 2)Face-to-face cognitive VRRS followed by at-home unstructured cognitive stimulation	Face-to-face cognitive treatment as usual	16 weeks	49	Cognitive training: Face-to-face and home-based VRRS treatment had tasks related to memory, attention and executive functions, and visuospatial abilities		Improvement in memory and visuo-constructional abilities in patients who completed face-to-face VRRS treatment compared to face-to-face treatment as usual. Memory: *p* = 0.047 Spatial orientation: *p* = 0.009			
Lee at al. (2020) [15]	Volunteers 60 or older with MCI recruited from South Korean hospital	Home-based robot cognitive intervention	No cognitive intervention	4 weeks	46	Cognitive training: Programs for memory training, frontal executive function, visuospatial function, etc.		Only improvement in working memory Robot group vs. control group after 4 weeks: Spatial working memory: *p* = 0.037 Paired associates learning: *p* = 0.744 Rapid visual information processing: *p* = 0.366			
Yu et al. (2019) [16]	Older adults from elderly community home in Hong Kong and their family caregivers	Dyadic strength-based empowerment program (D-StEP-MCI program) consisted of group-based session to the people with MCI (PwMCI), home-based dyadic sessions, and telephone follow-up	Usual care provided by elderly community	14 weeks	103 patient-caregiver dyads	Stress reduction: Stress adaptation strategies Cognitive training: Strength-based and empowerment program		Significantly improved the cognitive function and subjective memory of the PwMCI D-StEP-MCI group had improvement in MMSE score compared to control: *p* < 0.001			
Dannhauser et al. (2014) [17]	MCI patients from two local memory clinics	Thinkingfit program (three-component intervention: physical activity, group-based cognitive stimulation (GCST), individual cognitive stimulation (ICST))	Participants served as their own controls	12 weeks	67	Physical activity: Unsupervised walking from home or upright exercise bike Cognitive training: GCST: 8 participant groups engaged in adult education classes in arts and crafts ICST: Luminosity program consisting of games and puzzles either at community center or at home		Improvement or stability of cognitive outcomes following the intervention Working memory: *p* < 0.05			
Boespflug et al. (2018) [18]	Participants 68 years and older recruited from region in and around Ohio, USA with print notices	Blueberry supplementation	Placebo-controlled group	16 weeks	16	Diet: Powder prepared from whole freeze-dried blueberries		Enhanced neural response during working memory tasks No clear indication of WM enhancement Increased BOLD activation in the left pre-central gyrus, left middle frontal gyrus, and left inferior parietal lobe during WM load conditions (*p* < 0.01)			
Bo et al. (2017) [19]	MCI individuals aged 60 or older in Chinese elderly	n-3 polyunsaturated fatty acids supplementation	Placebo-controlled group	6 months	86	Diet: n-3 polyunsaturated fatty acids, specifically eicosapentaenoic acid (EPA) and docosahexaenoic acid (DHA)		480 mg/day DHA and 720 mg/day EPA could improve working memory, perceptual speed, and space imagery efficiency Improved total BCAT scores, perceptual speed, space imagery efficiency, and working memory (*p* < 0.01), but not with mental arithmetic efficiency or recognition memory (*p* > 0.05).			
Desideri et al. (2012) [20]	MCI individuals recruited in Italy	Once a day dairy-based cocoa drink contained coca flavanols at 1) high level 2) intermediate level 3) low level	No control group	8 weeks	90	Diet: High flavanol levels (990 mg flavanols/serving) Intermediate flavanol levels (520 mg flavanols/serving) Low flavanol levels (45 mg flavanols/serving)		Improved cognitive performance with regular coca flavanol consumption The time required to complete Trail Making Test A and Trail Making Test B was significantly (*p* < 0.05) lower in subjects assigned to high flavanols and intermediate flavanols compared with those assigned to low flavanols Verbal fluency test score was significantly (*p* < 0.05) better in subjects assigned to high flavanols in comparison with those assigned to low flavanols			
Fortier et al. (2020) [21]	Individuals aged 55 or older with MCI in Canada	Ketogenic drink containing medium chain triglyceride	Placebo	6 months	83	Diet: 15 g twice/day of ketogenic drink with medium chain triglyceride		Verbal fluency, free and cued recall improved in experimental vs. placebo group Free and cued recall: *p* = 0.047 Verbal fluency: *p* = 0.024 Boston Naming Test: *p* = 0.033 Trail-Making Test: *p* = 0.017			
Fotuhi et al. (2016) [22]	Elderly patients with MCI who presented to a community neurology practice in Maryland (USA)	Multicomponent “Brain Fitness Program”	No control group	12 weeks	127	Physical activity: Physical fitness education Diet: Education about Mediterranean diets and omega-3 supplements Stress reduction: Meditation and stress reduction training Cognitive training: Computer-based cognitive training program with games to improve memory, attention, etc. Neurofeedback therapy to improve memory, attention, etc.		Improvements in attention, concentration, executive function, among others. 84% of the patients experienced statistically significant improvements in their cognitive function (*p* < 0.05)			
Hwang et al. (2019) [23]	Physically healthy individuals with MCI recruited from hospitals in Korea	Lactobacillus plantarum C29-fermented soybean (DW2009) as a nutritional supplement	Placebo	12 weeks	100	Diet: 800 mg/day of DW2009		Enhanced cognitive function Combined cognitive functions: *p* = 0.02 Attention domain: *p* = 0.02			
Köbe et al. (2016) [24]	Patients 60–80 years recruited in Germany	Omega-3 FA supplementation, aerobic exercise and cognitive stimulation (target intervention)	Omega-3 FA supplementation and non-aerobic exercise (control intervention)	6 months	22	Physical activity: Target intervention: Aerobic training 45 min twice a week Control intervention: Non-aerobic training consisting of stretching and toning Diet: Omega-3 FA (2.2 g/day) in both target and control intervention groups Cognitive training: Target intervention: cognitive stimulation using AKTIVA program and advice on performing cognitively stimulating activities at home		No significant differences in cognitive performance between groups Executive function: *p* = 0.160 Memory: *p* = 0.635 Sensorimotor speed: *p* = 0.345 Attention: *p* = 0.656			
Rondanelli et al. (2012) [25]	Patients 70 or older with MCI recruited in Pavia (Italy)	Supplement their diet with an oily emulsion of docosahexaenoic acid (DHA)-phospholipids containing melatonin and tryptophan	Placebo	12 weeks	25	Diet: 2 capsules once a day: DHA 720 mg, EPA 286 mg, vitamin E 16 mg, soy phospholipids 160 mg (phosphatidylinositol, phosphatidylcholine, and phosphatidylethanolamine), tryptophan 95 mg, and melatonin 5 mg		Significant improvements in several measures of cognitive function compared to placebo MMSE: *p* < 0.001 Semantic verbal fluency: *p* < 0.06			
Ma et al. (2018) [26]	Patients 76–87 with MCI in Korea	Home-based board game program	Participants served as their own controls	4 weeks	3	Cognitive training: assortment of board games were played 3 times a week, 40 min per session		MMSE-K and MoCa-K results after the program showed a positive change in attention *p*-values were not provided			
Lim et al. (2023) [27]	Patients 65 years or older with MCI in Korea	Cognitive rehabilitation training using a serious game (Brain Talk™)	No cognitive intervention	4 weeks	24	Cognitive training: home-based serious game that involves several cognitive domains		Immediate improvement in K-MMSE, K-MoCA, and SVFT scores compared to control group, and the effects of continued after 1 month MMSE-K: *p* = 0.001 MoCA-K: *p* = 0.01 SVFT: *p* = 0.044			
Baik et al. (2024) [28]	Community-dwelling adults 55 or older with MCI in Korea	Home-based computerized cognitive training	No intervention	8 weeks	50	Cognitive training: home-based computerized cognitive training program called Neuro-World		The efficacy of the program was demonstrated through evaluations of language, memory, and executive function MoCA: *p* < 0.01 Digit span tests to assess memory and attention: *p* = 0.000 Semantic word fluency test: *p* = 0.004			

### Multi-component interventions

Multi-component interventions were defined as including two or more lifestyle modifications from the following categories: diet, physical activity, stress reduction, and cognitive training. The database search found five studies that included multi-component interventions [10, 16, 17, 22, 24]. Only two studies were identified that included stress reduction training as a method to improve cognitive function, and they were both part of a larger multi-component study [16, 22].

Out of the total twenty interventions analyzed, nineteen reported significant improvements in cognitive function between the experimental and control groups for at least one aspect of cognition. Four of these were multicomponent interventions [10, 16, 17, 22]. Yu et al. reported that participants in a strength-based and empowerment program, which includes cognitive and stress adaptation training, had improvements in their Mini-Mental State Examination (MMSE) scores compared to the control group of usual care provided by the elderly (*p* < 0.001) [16]. Dannhauser et al. found that a three-component intervention composed of physical activity, group-based cognitive stimulation, and individual cognitive stimulation resulted in significant improvements in working memory [17]. A four-component intervention by Fotuhi et al. found that physical fitness education, education about Mediterranean diets and omega-3 supplements, meditation and stress reduction training, and cognitive training programs resulted in significant improvements in cognitive function (*p* < 0.05) [22]. Horie et al. studied the impact of physical activity encouragement and nutrition counselling and found improvements in global cognition compared to the control group (*p* < 0.0001) [10].

### Single-component interventions

The fifteen single-component interventions all found significant improvements in at least one area of cognitive function, in which six studied the effect of diet, two studied the impact of physical activity, and seven analyzed the effect of cognitive training. Manenti et al. studied the effect of face-to-face cognitive virtual reality rehabilitation system and cognitive stimulation on cognitive function and found that they had improvements in memory, language and visuo-constructional abilities compared to face-to-face treatment as usual [14]. Lee et al. found that a home-based robot cognitive intervention resulted in improvements in spatial working memory (*p* = 0.037) but not in paired associates learning (*p* = 0.744) or rapid visual information processing (*p* = 0.0366) [15]. Frain et al. utilized a computerized cognitive training program and found significant improvements in total Montreal Cognitive Assessment (MoCA) scores compared to control (*p* = 0.04) [9]. Jeong et al. studied a group-based cognitive intervention (GCI) and home-based cognitive intervention (HCI) and found that compared to the control which received neither intervention, subjects in both intervention groups had improvements in cognition, with the benefits of cognitive intervention persisting for at least another 6 months after the study (*p* = 0.01 and *p* = 0.02) [11]. Sungkarat et al. found that tai chi exercises helped improve logical memory (*p* = 0.006) [12]. Suzuki et al. also studied physical activity and found that a structured exercise program had improvements in logical memory of immediate recall (*p* = 0.03) and letter verbal fluency test (*p* = 0.02) [13]. Boespflug et al. studied the effect of blueberry diet supplementation on blood oxygen level-dependent (BOLD) signal on neural activity. They found increased BOLD activation in the left interior parietal lobe, left pre-central gyrus, and left middle frontal gyrus during working memory load conditions (*p* < 0.01) [18]. Bo et al. studied n-3 polyunsaturated fatty acids supplementation to the diet. Participants demonstrated improvements in multiple areas of cognition, specifically total Basic Cognitive Aptitude Tests (BCAT) scores, space imagery efficiency, working memory, and perceptual speed (*p* < 0.01). Desideri et al. found that the time required to complete Trail Making Test A and B was significantly lower in subjects assigned to drinking coca drinks with high or intermediate flavanol levels (*p* < 0.05) compared to those assigned to low flavanols [20]. Fortier et al. found that subjects who drank a ketogenic drink with medium chain triglycerides had improvements in free and cued recall (*p* = 0.047) and verbal fluency (*p* = 0.033) [21]. Hwang et al. studied the effect of Lactobacillus plantarum C29-fermented soybean (DW2009) as a nutritional supplement on cognition and found improvements in combined cognitive functions and the attention domain (*p* = 0.02) in the experimental group compared to control group [23]. Rondanelli et al. found that those who supplemented their diet with an oily emulsion of docosahexaenoic acid (DHA)-phospholipids containing melatonin and tryptophan had improvements in MMSE scores (*p* < 0.001) and semantic verbal fluency (*p* < 0.06) [25]. Ma et al. studied the effect of playing an assortment of board games on cognitive function and found improvements in attention as demonstrated by increases in MMSE-K and MoCA-K scores [26]. Lim et al. found that individuals participating in cognitive training through a serious game demonstrated immediate improvement in MMSE-K (*p* = 0.001), MoCA-K (*p* = 0.01), and SVFT scores (*p* = 0.044), with effects continuing one month post-intervention [27]. Baik et al. analyzed the effect of a home-based computerized cognitive training intervention called Neuro-World, and determined there were improvements in MoCA scores (*p* < 0.01), digit span test results (*p* = 0.000), and semantic word fluency test results (*p* = 0.004) [28].

Four studies reported no significant differences in cognitive performance between the experimental and control groups for at least one aspect of cognition, of which only Köbe et al.’s study was multi-component [15, 18, 19, 24]. One study’s experimental group had omega-3 fatty acid (FA) supplementation, aerobic exercise, and cognitive stimulation whereas the control group had omega-3 FA supplementation and non-aerobic exercise [24]. No significant changes in memory (*p* = 0.635), executive function (*p* = 0.160), sensorimotor speed (*p* = 0.345) and attention (*p* = 0.656) were found in both groups after correction for multiple-comparisons [24]. Boespflug et al. studied the effect of blueberry diet supplementation on blood oxygen level-dependent (BOLD) signal on neural activity. There was no clear evidence of an enhancement in working memory post-intervention [18]. Another study analyzed the impact of a home-based robot cognitive intervention versus a control group with no cognitive intervention [15]. After the 4-week intervention, there was no significant difference between the two groups in paired associates learning (*p* = 0.744) or rapid visual information processing (*p* = 0.366) [15]. Bo et al. studied n-3 polyunsaturated fatty acids supplementation to the diet. There was no significant improvement in recognition memory or mental arithmetic efficiency (*p* > 0.05) [19].

## Discussion

In our comprehensive review, we identified five multi-component and fifteen single-component home-based intervention studies from our twenty total eligible research papers. Of the five multi-component studies, four indicated the effectiveness of home-based lifestyle interventions in enhancing cognitive function within at least one cognitive domain, whereas only one demonstrated no significant change in at least one aspect of cognition after the intervention. All the fifteen single-component interventions found improvements in cognitive function within at least one cognitive domain. Six studied the effect of diet, two studied the impact of physical activity, and seven analyzed the effect of cognitive training.

There is a dearth of multi-component lifestyle interventions that fit within our search criteria. Research examining the impact of stress reduction training is particularly scarce and was not analyzed as single components; rather, they were included only in two multi-component studies. Four studies demonstrated no significant change in at least one aspect of cognition after the intervention, with only one being multi-component [15, 18, 19, 24]. Of these four studies, two focused solely on dietary interventions, one focused solely on cognitive interventions, and one studied a multidomain intervention consisting of dietary, cognitive, and physical activity interventions. It is worth noting that among the two dietary interventions that concluded no significant change in at least one aspect of cognition, there were still improvements in other areas of cognitive function, specifically in neural responses, working memory, space imagery efficiency, and perceptual speed [18, 19].

Most of the studies had web-based components in addition to home-based, such as the use of robots or online applications. The use of web-based health interventions in chronic disease management has revolutionized the field of healthcare. These interventions offer several advantages over traditional interventions, particularly in terms of improving accessibility, convenience, and user engagement. With virtual health interventions, patients can access their healthcare from anywhere and at any time, which can lead to better adherence to treatment and improved health outcomes. Moreover, these interventions offer the potential for continuous monitoring and feedback, which can help to optimize the intervention and provide personalized care. These advantages are particularly relevant in the context of MCI as it often affects older adults, who may have limited mobility and may face challenges in accessing traditional healthcare settings. By providing interventions through mobile devices, virtual interventions can overcome these barriers and provide older adults with a convenient and accessible way to manage their cognitive impairment.

It is important to note that the penetration of mobile technology among older adults may be lower compared to younger populations. However, over the past decade and with COVID-19 pandemic, an increasing number of elderly individuals have used telehealth and mobile devices from their home for their healthcare needs [29]. Thus, it is essential to assess the penetration of mobile-based interventions among older adults and to ensure that the interventions are designed in a way that is accessible and user-friendly for this population. Additionally, it is important to consider the unique challenges faced by older adults in terms of cognitive and physical abilities, and to design interventions that are tailored to their needs.

Although limited, there are existing systematic reviews that detail the effect of lifestyle interventions on cognition of older adults with MCI [4]. However, these studies did not include RCTs that are specifically home-based. Thus, more research is needed to analyse how home-based interventions can impact cognition and address this gap in the scientific literature. This is particularly important for the very elderly as they frequently have barriers for transportation and the ones living in rural areas with limited access to healthcare facilities for in-person interventions.

Our systematic review is dependent on the quality of the contributing studies and has a few limitations. Some of the studies focused only on MCI participants while two other studies targeted MCI individuals with specific clinical circumstances of HIV or obesity. The results from these two studies may not be as applicable to individuals with MCI in general. Furthermore, a few of the studies (*n* = 6) had a smaller sample size of 25 people or less or were of short duration of 4 weeks or less (*n* = 4), which could have affected the reliability of the results.

## Conclusion

Our systematic review suggests that home-based lifestyle interventions focusing on diet, physical activity, stress reduction, and cognitive training can help combat cognitive decline and improve brain function. Since MCI is often a precursor to more serious forms of dementia, there is a need for effective lifestyle interventions that can be conveniently performed from home via telemedicine or other accessible formats. More robust RCTs with larger sample sizes and longer duration are needed to provide stronger evidence of the effectiveness of these interventions on specific cognitive domains and which components of lifestyle are most beneficial. Home-based interventions have the potential to slow cognitive decline and provide access to lifestyle support for the growing elderly population in US and globally.

### Electronic supplementary material

Below is the link to the electronic supplementary material.

Supplementary Material 1

## Data Availability

The datasets used and analyzed during the current study are available from the corresponding author on reasonable request.

## Abbreviations

aMCI: amnestic mild cognitive impairment
BCAT: Basic Cognitive Aptitude Tests
BOLD: blood oxygen level-dependent
DHA: docosahexaenoic acid
FA: fatty acid
GCI: group-based cognitive intervention
HCI: home-based cognitive intervention
MCI: Mild Cognitive Impairment
MMSE: Mini-Mental State Examination
MMSE: K-Mini-Mental State Examination Korean version
MoCA: Montreal Cognitive Assessment
MoCA: K-Montreal Cognitive Assessment Korean version
RCT: Randomized controlled trial
SVFT: Sematic Verbal Fluency Task
